# Cancer Cases Referral system in Nepal

**DOI:** 10.3126/nje.v8i4.23877

**Published:** 2018-12-31

**Authors:** Krishna Kanta Poudel, Deborah Sims, Dianne Morris, Prakash Raj Neupane, Anjani Kumar Jha, Nirmal Lamichhane, Ganga Sapkota, Dipendra Kumar Mallik, Zhibi Huang, Janaki Kharel Poudel, Elisabete Weiderpass

**Affiliations:** 1 Faculty of Health, University of Technology, Sydney; 2 Faculty of Health, University of Technology Sydney, PO Box 123 Broadway NSW 2007; 3 Centre for Midwifery, Child and Family Health (CMCFH) Faculty of Health | University of Technology Sydney Building 10, Level 7, 235 Jones St, Ultimo NSW 2007; 4 Head of Surgical Oncology Department, Bhakatapur Cancer Hospital, Nepal; 5 Chairman, Nepal Health Research Council Kathmandu, Nepal; 6 Deputy Director, B P Koirala Memorial Cancer Hospital, Chitwan, Nepal; 7 Junior Consultant B P Koirala Memorial Cancer Hospital, Chitwan, Nepal; 8 Medical Officer, B P Koirala Memorial Cancer Hospital, Chitwan, Nepal; 9 Department of Epidemiology and Biostatistics, School of Public Health, Guangxi Medical University, China; 10 Community Health Campaign, Chitwan, Nepal; 11 International Agency for Research on Cancer, World Health Organization, 150 cours Albert Thomas, 69372 Lyon CEDEX 08, France

**Keywords:** cancer, epidemiology, referral, Nepal

## Abstract

The burden of cancer is estimated to be increasing in Nepal, whilst the country lacks national established guidelines or protocols for referral of cancer cases. Cancer patients are presenting many different health facilities throughout the country. In rural areas almost all cancer patients have their first diagnosis when visiting a health assistant or nurse at their nearest primary health care delivery service. If cancer is suspected, health care assistants or nurses will refer the patient to a medical doctor at the primary health centre, or refer the patient directly to the cancer treatment centre or oncology department of the closest hospital. Patients from urban areas will usually be seen for the first time by a medical doctor initially and then referred to either the cancer treatment centre or oncology department of the hospital. Both in rural and urban areas the referral for treatment is determined by both the patients’ capacity to pay for treatment own healthcare, as well as their geographical location (i.e. availability and accessibility of cancer treatment services.

## Introduction

Nepal is situated between two large countries - China and India. These neighbouring countries have information systems in place including population based cancer registries, which is not the case in Nepal [1]. However, between 2003 to 2012 seven hospital-based cancer registries (HBCRs) were initiated in Nepal, [2,3,4,5,6] at:

- B P Koirala Memorial Cancer Hospital
- Bhaktapur Cancer Hospital
- Bir Hospital
- TU Teaching Hospital
- Kanti Children Hospital
- B P Koirala Institute of Health Science
- Manipal Teaching Hospital

A national cancer registry programme has been initiated in Nepal in 2003, which is in turn based on-based cancer registries [6].This initiative includes information from the registries above plus the following: [4]

- Shree Birendra Hospital
- Civil Service Hospital
- Patan Hospital
- Paropakar Maternity and Women Hospital
- Nepalgunj Teaching Hospital

Based on data retrieved from Nepalese hospital-based-cancer registries from 2003 to 2012 the total number of new cancer cases reported in the hospital-based registries listed above was 55,931 [3]. A 2013 report found that the number of new cancer cases in both sexes was 8,729 [4]. However, this is probably a large underestimation of the number of cancer cases. Globocan estimated that people newly diagnosed with cancer per year in both sexes in Nepal in year 2018 to be 26,184 [7]. Hospital-based cancer registration (HBCRs) remains incomplete for several reasons, including lack of access to health care due to financial constraints, which hampers diagnosis and care. Moreover, there is lack of awareness of a cancer as a disease in general, and that it can be prevented, diagnosed early, treated, and in many cases cured. Many cancer patients in Nepal receive no proper diagnosis and treatment, and die at home without palliative care [8].

Reports from the hospital-based registries in Nepal had indicate that lung cancer was the common cancer in men followed by stomach and larynx cancers. In women cervix cancer was the major cancer followed by breast and ovarian cancer from 2003 to 2013 [2, 3, 4, 5, 6].

Some studies indicated an increasing trend of cancer incidence in both sexes and all ages in Nepal between 2003 to 2013 [2, 3, 4, 5]. One **s**tudy also predicted that cancer incidence would continue to increase in both sexes from 13.9 in 2003 to 39.6 per 100,000 by 2020 [9]. Table 1 shows the estimation of crude incidence from the data of seven HBCRs between 2003 to 2020 in Nepal (Table 1) [9].

### Cancer treatment centres in Nepal

B. P. Koirala Memorial Cancer Hospital (BPKMCH) was established in 1992 with the support of Chinese government; the institutional aims are research, prevention and control of cancer in Nepal. BPKMCH was the first tertiary level cancer hospital in Nepal delivering all types of cancer treatment services, such as surgical oncology, medical oncology, radiation oncology, pathology services, radio diagnosis imaging and nuclear medicine services [10]. Published reports show that BPKMCH covered the largest number of new and old cancer cases throughout the country from 2003 to 2017 [10, 11]. As the number of new cancer cases increased [2,3,4,5,9], the government and private sector also established new cancer hospitals in different parts of the country. They are listed below.

- Kathmandu Cancer Centre
- Bhaktapur Cancer Hospital
- Nepal Cancer Hospital and Research Centre
- Suhil Koirala Prakhar Cancer Hospital

### Cancer Patients Referral system in Nepal

Generally most patients visit the nearest health service delivery organisation for initial health presentation. As there are no established guidelines or protocols for referral of cancer patients in Nepal, patients may visit any health delivery organisation. Patients from rural and remote areas usually receive medical assistance from health assistants or other health care staff. In urban areas, patient usually have direct access to physicians and treatment centres. If the health assistant or doctor at rural areas are unsure of the diagnosis or suspect a cancer diagnosis, patients are usually referred to a specialist, or directly to a cancer treatment centre or oncology department of any hospital.

**Figure 1 –:**
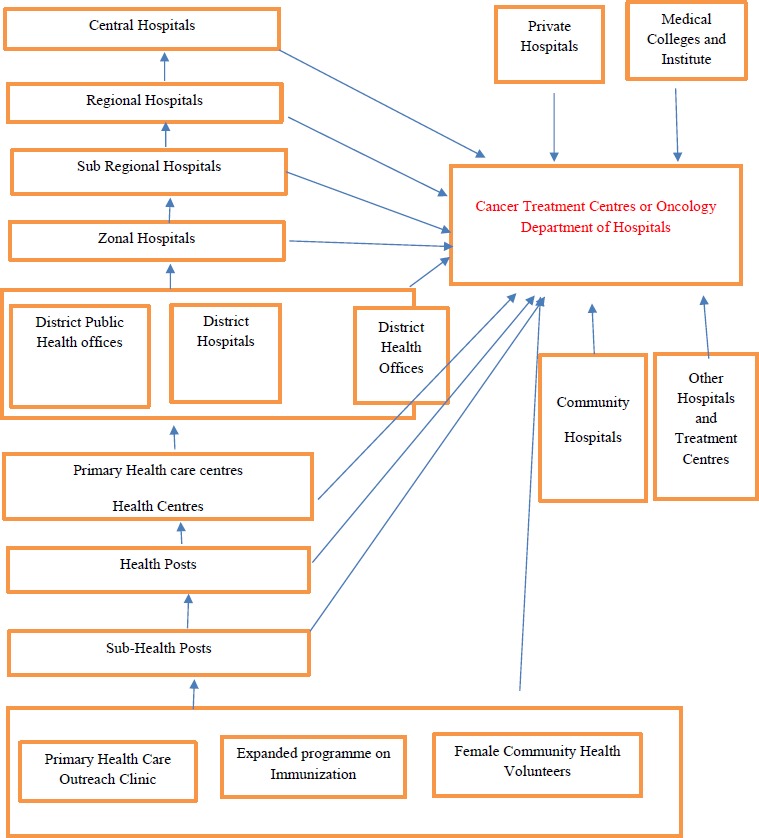
Cancer cases referral system in Nepal

Access to diagnosis and treatment depends on geographical proximity to health care facilities and by the capacity of the patient of funding his or her own healthcare. Due to lack of national health insurance scheme [12], patients bear all the financial burden of treatment. Since last decade, the Ministry of Health in Nepal is providing US $ 620 to support each individual who is diagnosed with cancer. This amount is directly transferred to the cancer institution in Nepal to cover the expenses of treatment such as surgery, radiotherapy, medications and diagnostic and follow up investigations [12]. Most patients receive treatment at the hospital which is nearest to their homes (Figure 1).

### Challenges for the research, prevention and control of cancer in Nepal

In Nepal there are a number of challenges for preventing, controlling and reporting cancer [8]. There are no population-based national records and cancer treatment facilities are inadequately resourced. Given the increasing numbers of patients, waiting time for treatment is long. Cancer mortality is increasing, and early detection programs are unavailable [8].

Piya and Acharya [12] presented the following challenges of cancer treatment and diagnosis in Nepal:

- Cost of treatment, absence of health insurance schemes, and ineffective health policies, requiring patients to bear financial burden of diagnosis and treatment.
- Lack of awareness about the prognosis of disease. As a consequence of a delay in the presentation of patients at hospital. There is an increase in the number of patient with advanced stages of cancer and thus morbidity and mortality.
- Inappropriate laws and regulations regarding the radiation, and lack of radiation oncology and nuclear medicine equipment, which are essential for the treatment of cancer.
- Limited capacity to import chemotherapy, making access to the drugs difficult.
- Developments of cellular and molecular diagnosis made elsewhere only reach Nepal slowly, if at all.
- Lack of trained human resources in cancer prevention, early detection, diagnosis and treatment.

To decrease the burden of cancer, the stakeholders and concerned authorities should implement public health policies aiming to increase health awareness and health education in general [8]. In addition, Nepal needs primary cancer prevention strategies, such full compliance to the framework against tobacco, as well as making vaccination against Human Papilloma Virus and Hepatitis B Virus universally available.

Cancer screening programmes for cervical cancer, breast cancer and bowel cancer need to be considered. Developing of cancer control and prevention policies are essential to decrease the cancer burden in Nepal. The establishment of new cancer centres should be considered. A national cancer institute should be established with diagnostic, treatment and research facilities, including cancer registry to record cancer incidence and prevalence and monitor mortality across the country to enable better management of the burden of cancer in Nepal [8].

## Figures and Tables

**Table 1:** Observed cancer incidence (2003-2012) and estimated incidence for (2013-2020) both gender in Nepal [9].

Year	Male crude incidence	Female crude incidence	Both sex crude incidence
2003	12.8	15.1	13.9
2004	17.0	18.3	17.7
2005	17.1	19.4	18.2
2006	19.0	21.1	20.0
2007	20.8	24.6	22.7
2008	21.6	25.9	23.8
2009	23.4	25.4	24.4
2010	24.8	27.8	26.3
2011	26.7	27.5	26.6
2012	25.3	28.1	26.7
2013	28.6	31.3	29.8
2014	30.0	32.7	31.2
2015	31.4	34.1	32.6
2016	32.8	35.6	34.0
2017	34.2	37.0	35.4
2018	35.6	38.5	36.8
2019	37.1	39.9	38.2
2020	38.5	41.4	39.6
